# The Early Phases of Ankylosing Spondylitis: Emerging Insights From Clinical and Basic Science

**DOI:** 10.3389/fimmu.2018.02668

**Published:** 2018-11-16

**Authors:** Abdulla Watad, Charlie Bridgewood, Tobias Russell, Helena Marzo-Ortega, Richard Cuthbert, Dennis McGonagle

**Affiliations:** ^1^Section of Musculoskeletal Disease, Leeds Institute of Molecular Medicine, University of Leeds, NIHR Leeds Musculoskeletal Biomedical Research Unit, Chapel Allerton Hospital, Leeds, United Kingdom; ^2^Department of Medicine “B”, Zabludowicz Center for Autoimmune Diseases, Sheba Medical Center, Tel-Hashomer, Ramat Gan, Israel; ^3^Sackler Faculty of Medicine, Tel-Aviv University, Tel-Aviv, Israel

**Keywords:** ankylosing spondylitis, spondyloarthritis, HLA-B27, enthesitis, microbiota, IL-17, IL-23, TNF-α

## Abstract

In our paper, we discuss how the early phases of ankylosing spondylitis (AS) are linked to peri-firbocartilagenous osteitis in the sacroiliac joint and entheseal bone related anchoring sites. This skeletal proclivity is linked to an abnormal immunological response to skeletal biomechanical stress and associated microdamage. A key event in the early stages of AS appears to be the association with subclinical Crohn's-like colitis with this gut inflammation being pivotal to the osteitis reaction. Whether this osteitis is consequent to non-specific intestinal innate immune activation or adaptive immune responses against specific microbiotal or self-antigens is unknown. Recurrent iritis is an HLA-B27 associated feature that may predate AS and pursues a course independent of joint involvement, and points toward the pivotal role of organ specific immunology over generalized systemic immune responses in disease expression. Human genetics and animal model studies strongly incriminate the IL23/17 axis and TNF-α in disease pathogenesis. Preliminary work shows a strong convergence of innate immune cells including type 3 innate lymphoid-cells (ILC3) and γδ T-cells in skin, gut, entheseal, and eye inflammation. Despite the HLA-B27 association, the role of adaptive immunity, especially CD8+ T-cells mediated responses remains unproven and alternative theories have been proposed. The emerging non-dependence of axial inflammation on IL-23 but dependence on IL-17A is an unexpected new twist that awaits full explanation. In this mini-review, we discuss the key events in the early stages of human AS from clinical and basic science aspects, which could be crucial for attempted disease prevention studies in at risk subjects.

## Introduction

Ankylosing spondylitis (AS) is the prototype of the inflammatory rheumatic diseases grouped under the term spondyloarthritis (SpA) and represents the end phenotype of the axial SpA group (1). The understanding of the male predominant, a late adolescent insidious onset of AS with spinal involvement, characteristic extra-articular manifestations, and post inflammatory new bone formation has remained relatively enigmatic (2). The pathogenesis of AS is not completely understood, but likely involves a complex interplay between genetic predisposition involving the human leucocyte antigen, namely HLA-B27, and environmental factors such as mechanical stress and the microbiome (2, 3). In the last decade, imaging as well as animal model studies have pointed toward enthesitis and associated osteitis as the primary pathological process in SpA including AS (4, 5) and we will thus focus on these early lesions.

The diagnosis of AS is determined according to the New York criteria with the earlier pre-radiographic phases of AS defined by the ASAS (Assessment of Spondylo-Arthritis International Society) (6, 7), thus, we will focus on these pre-radiographic phases since they may culminate in frank AS. Accordingly, this early phase of disease includes nrAxSpA which may be evident on MRI assessment and also includes the events that predate the MRI lesions. The lack of a gold standard measure for predicting AS such as AS-specific autoantibodies that may predate the radiological or clinical presentation of SpA makes the early disease phases virtually impossible to clearly discern, compared to humoral associated autoimmune diseases like rheumatoid arthritis (RA), or systemic lupus erythematosus (SLE) (8). Other factors contributing to the difficulty in studying the early phases of AS is the failure to recognize inflammatory back pain, and the absence of visible swelling and often normal inflammatory markers. The purpose of this perspective is to describe the events that lead to disease initiation in the skeleton and extra-skeletal sites in human AS.

## The initiation of AS disease and skeletal mechanics

**Figure 1:**
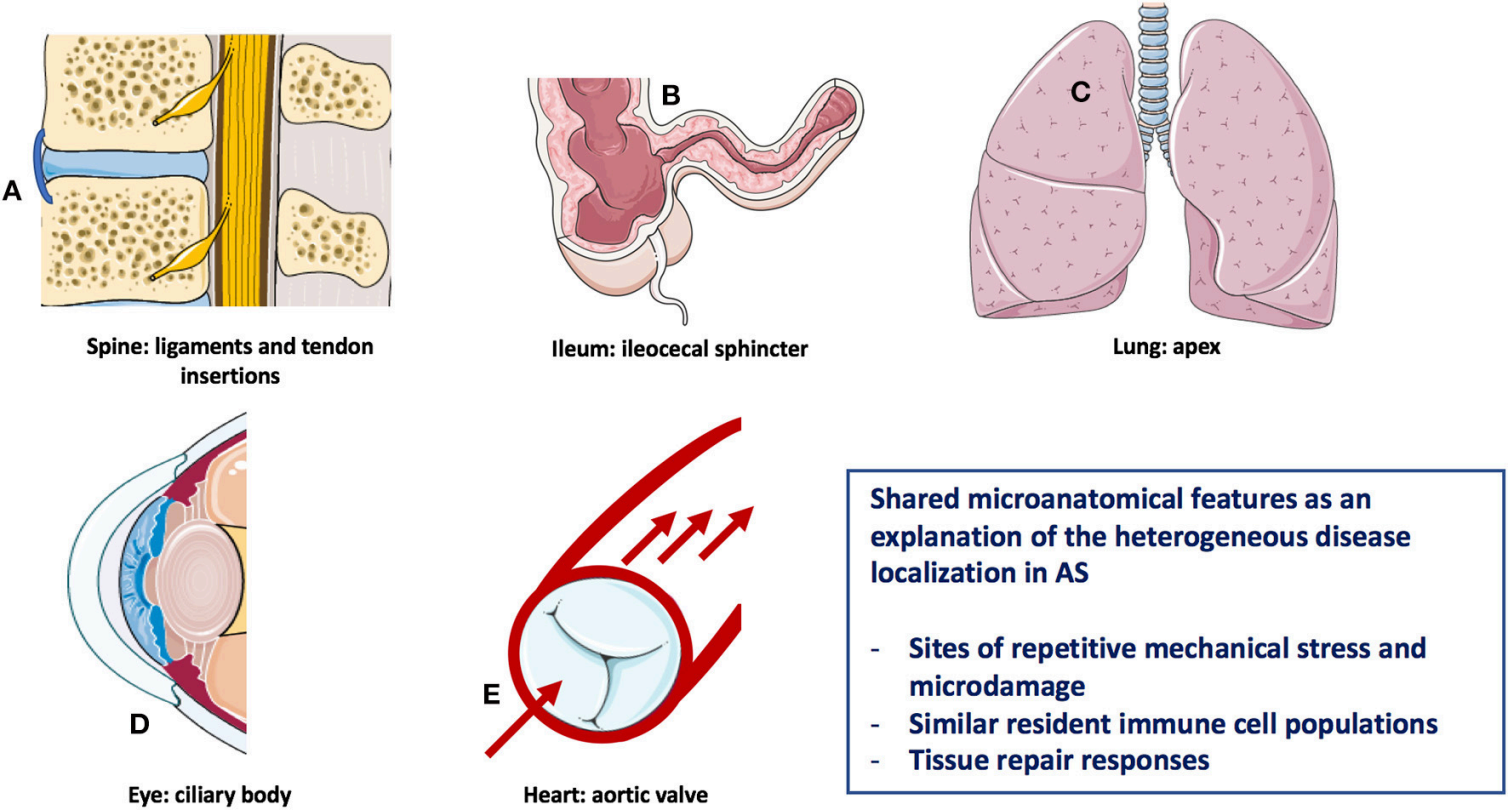
Heterogeneous AS disease localization sharing microanatomical features. Features of this figure are reproduced from https://smart.servier.com (Servier Medical Art by Servier is licensed under a Creative Commons Attribution 3.0 Unported License), and were changed in terms of shape and size.

Analogous to most of the autoimmune/autoinflammatory diseases (9), the pathogenesis of AS is multifactorial and results from a complex interplay between genetic predisposition and environmental triggers (10). The modern understanding of immune diseases classification that is especially relevant for the rheumatic disorders, and is predicated on the concept that disease may start in the primary and secondary lymphoid organs, this type being autoimmune in nature, or may start in the target tissues with tissue specific innate immune activation and then secondary adaptive immune responses leading to the clinical phenotype (11, 12). The pathological basis for AS related inflammation affecting the anterior uveal tract, the aortic root/valve, the lung apex, and enthesis organ structures including sites of bone adjacent to entheseal and synovio-entheseal fibrocartilages has been better conceptualized in relationship to common biomechanical factors rather than autoimmunity to a common antigen/autoantigen at these disparate sites (13) (Figure 1). The strong tropism for the SpA associated uveitis to occur in the “moving parts” of the anterior uveal tract and the link between sonographically determined subclinical lower limb enthesopathy and anterior uveitis points to the importance of anterior uveal track biomechanics (14).

A key aspect of understanding the diverse pattern of organ involvement in AS relates to the fact that mechanically stressed disease prone sites are subject to recurrent tissue microdamage (15, 16). For example, the aortic root and lung apex are both recognized as target tissues in AS with these regions of the body being subject to bleb formation and valvular damage, respectively in other settings (16) (Figure 1). In the skeleton in AS, there is a strong evidence that site specific localization of disease is dependent on bone stressing. Clues to the cardinal role of skeletal biomechanics come from pediatric HLA-B27-related arthritis which has a propensity of midfoot and lower limb enthesitis and oligoarthritis (17). With increasing age and change in BMI and muscle structure, pediatric disease migration with more topographic sacroiliac joints (SIJ), and subsequent spinal involvement occurs, which mirrors the more common pattern of young adult onset disease (17). This physical stressing concept may also apply to the terminal ileum and bowel where the hostile environment may be associated with spontaneously healing apthous ulcerations in normal subjects (18) (Figure 1).

**Figure 2:**
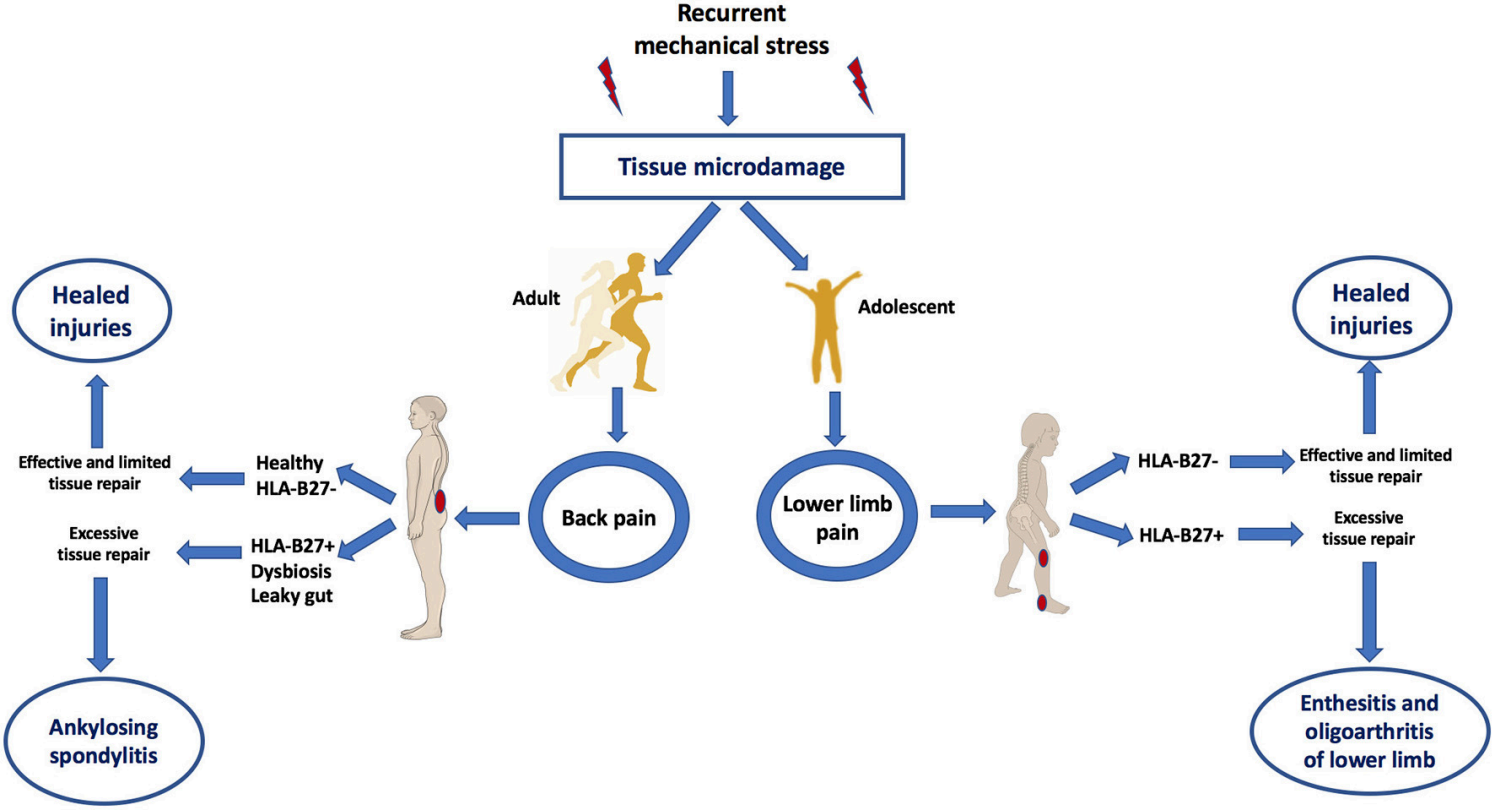
Mechanical stress as a trigger of tissue microdamage and back pain and its consequences in those HLA-B27+ subjects with dysbyosis compared to healthy subjects. Patients with AS have what is termed “inflammatory back pain” at the earliest stages of disease. However, skeletal microdamage that is viewed as “non-inflammatory” has an initial inflammatory component to initiate removal of microdebris and tissue repair. Recognition that both early AS and non-AS back pain shares the commonalities of skeletal biomechanical stress, microdamage and microinflammation for tissue repair helps explain why it is very difficult in many subjects to accurately differentiate between inflammatory and “non-inflammatory” disease. Bone stressing reactions in AS and its juvenile genetically related equivalent occurs at sites of complex compression and shearing and tensile forces. Innate immune dysregulation at such sites contributed to by gut barrier dysfunction may result in entheseal immune activation. Subjects with HLA-B27 gene, dysbyosis and leaky gut are associated with adaptive immune activation and the characteristic early disease MRI phenotype of osteitis. Features of this figure are reproduced from https://smart.servier.com (Servier Medical Art by Servier is licensed under a Creative Commons Attribution 3.0 Unported License), and were changed in terms of shape and size.

According to this biomechanical model of early AS, it would be predicted that the target sites of AS including the SIJ and axial and peripheral entheses would also exhibit similar MRI patterns of bone oedema on skeletal stressing in subjects without AS (13). Indeed this was first elegantly demonstrated in 2005 whereupon intense physical training in elite recruits developed bone stressing responses around the skeleton, including SIJs and in many cases, the lesions were asymptomatic (19). Interestingly, these “injuries” healed or remained asymptomatic, despite the continuation of intensive physical activity. A more recent Belgian study focussed on the SIJ and clearly showed transient physical stress related bone marrow oedema patterns that were initially reported as being a key feature of AS (20). Another recent paper showed inferior SIJ bone oedema lesions were not uncommon in elite athletes (21). Therefore, recurrent mechanical stress triggers tissue microdamage and repair processes that occur exactly in the same target sites as AS (Figure 2).

The question arises regarding the mechanism of new bone formation in AS (a relatively late feature of disease) but not in normal subjects that ostensibly experience the same biophysical stressing. A genetic basis related to tissue remodeling differences in AS is not compelling. Genetic polymorphisms in prostaglandin receptor might be directly linked to an aberrant new bone formation or downstream effect of IL-23 signaling on IL-22 which in turn is known to drive skeletal stem cells (22). Alternatively, the repair responses might be directly linked to the magnitude of the preceding inflammation which in turn is linked to the complex interplay between genetic susceptibility, dysbiosis, and leaky gut to induce in SpA in comparison to healthy subjects, wherein the latter a physiological level healing of the tissue microdamage occurs.

Tissue microdamage has been clearly histologically demonstrated in the spines of otherwise healthy subjects from the third decade of age onwards with lesions having the same topography of commonly viewed areas of MRI bone oedema in AS (23). Ultrasound studies have also pointed toward damage in normal entheses from young subjects without SpA (24). Several studies show histological damage in normal spinal and peripheral entheses in subjects without SpA (25) and also microscopic inflammation in these tissues (26). Hence, there is good evidence for microdamage and associated healing responses in AS prone sites.

## The role of enthesis tissue anatomy in early stages of AS

**Figure 3:**
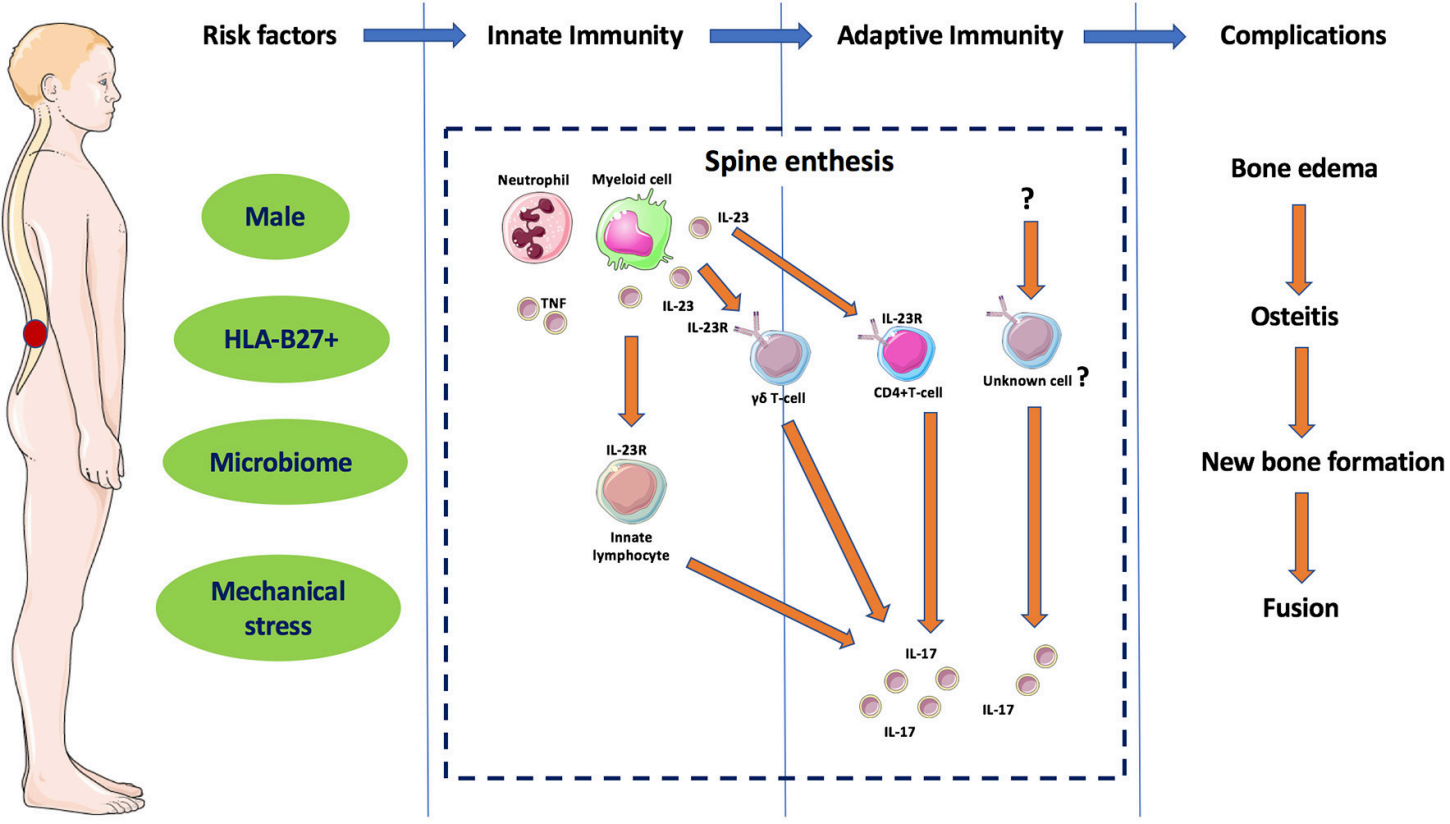
Different phases of the immunopathogenesis of AS. The interaction between different components considered as risk factors for AS leads to innate immunity dysregulation in the spinal entheses and other sub-fibrocartilagenous bone. Local secretion if IL-23 and activation of ILC3 and in later phase to adaptive immunity dysregulation with activation of IL-23R positive immune cells such as γδ T-cells and other unknown cells releasing IL-17 that results in remodeling effects including bone oedema, osteitis, new bone formation and in some cases spine fusion. Features of this figure are reproduced from https://smart.servier.com (Servier Medical Art by Servier is licensed under a Creative Commons Attribution 3.0 Unported License), and were changed in terms of shape and size.

From a pathological perspective, AS skeletal involvement has a strong propensity for peri-fibrocartilage osteitis in early SIJ involvement and in later spinal involvement. Fibrocartilage development dynamically occurs at sites of complex patterns of skeletal stressing, including tension, and compression that occurs at several synovial joints (SIJ, sternoclavicular, manubriosternal and acromioclavicular joints) that are positioned perpendicular to the ground and also entheses which share similar patterns of biomechanical stress (15). Biomechanical testing studies have shown that complex patterns of bone stressing are associated with bone failure, so it is likely that this is key at sites of simultaneous compressing and shearing forces on the skeleton (27). The particular proclivity for AS peri-fibrocartilage related osteitis in the SIJs, wrap around tendons and in the bone at entheseal anchorage sites has been reviewed previously (15, 28). Thus, peri-fibrocartilageneous bone and entheses represent a primary site and tissue target where innate and adaptive reactions occur initially as a repair process, but in later phases can result in remodeling effects including bone oedema, osteitis, new bone formation, and in some cases spine fusion in those genetically predisposed (Figure 3). Such predispositions include carriage of HLA-B27 gene with associated abnormal gut permeability, with a ramped up inflammatory response with a subsequent post inflammation excessive repair reaction that may ultimately culminate in ankylosis (1).

## Insight and lessons learnt from imaging studies in early phases of AS

It is known from MRI studies that carriage of the HLA-B27 gene is a risk factor for the extent and chronicity of bone marrow oedema lesions, histologically classed as osteitis, in the SIJ in AS (29). Likewise, it has been shown that HLA-B27+ axial PsA subjects have a much greater degree of MRI determined axial osteitis compared to axial disease in HLA-B27- subjects (30). Furthermore, the severity of peripheral skeletal involvement in SpA related heel disease is linked to a carriage of the HLA-B27 gene which is of a greater magnitude than the mechanically driven disease (29). Patients with early inflammatory back pain and a combination of severe sacroiliitis and HLA-B27 positivity are at higher risk for development of AS, compared with mild or no sacroiliitis, regardless of HLA-B27 status (31). The role of adaptive immunity in the clinical expression of AS in the trabecular bone seems likely based on disease MHC-I associations, most notably HLA-B27 but also HLA-B40, in addition to several other genes involved in T-cell biology (12, 32).

## The importance of the gut in early AS

Up to 10% of AS patients are reported to have IBD and 70% shows signs which indicate subclinical intestinal inflammation (33). From genome wide association studies (GWAS), it is evident that over 10% of the gene pathways are shared between IBD and AS (34). An important recent study showed a clear link between the severity of subclinical gut inflammation and MRI determined SIJ involvement (20). It has long been suggested that intrinsic barrier dysfunction permits non-specific innate immune activation with systemic translocation of bacterial adjuvants (16). In AS, the tight junctions between intestinal epithelial cells are prone to increased permeability and this is termed a leaky gut (35, 36). Several molecules increased in AS may point toward the idea of a leaky gut such as LPS-binding protein, fatty acid binding protein and zonulin (37–39). In recent times, specific alterations into the composition of the gut microbiota, rather than the non-specific innate activation theory have emerged and have been associated with a range of immune mediated disorders (39). It has been postulated that carrying HLA-B27 is predisposing for gut dysbiosis followed by a leaky gut and subsequent systemic entrance of microbial antigens and adjuvants, which may act as a trigger for enthesitis. These adjuvants may activate entheseal stromal and immune resident cell populations leading to the activation of the IL-23/IL-17 axis, secretion of pro-inflammatory cytokines resulting in enthesitis, osteitis and joint local inflammation (40, 41).

## Genetic influences in early AS

The genetics of AS including the striking HLA-B27 is well recognized and reviewed elsewhere and is only briefly discussed here (42, 43). The carriage of HLA-B27 gene in AS is well-established and varies according to the populations (44–46) with other MHC-I antigens, most notably HLA-B40 being linked to disease (47). Up to 167 subtypes of HLA-B27 have been reported, with those not associated with AS typically having structural changes within the amino acid peptide binding groove (48), which may support the concept of antigen presentation in disease. Less than 5% of those positive for HLA-B27 ever develop AS, pointing toward other key genes and other factors influencing early disease susceptibility (49).

GWAS studies have also shown that SNPs in ERAP-1 in epistatic interaction with HLA-B27 are common in AS (47, 50). ERAP-1 interactions with HLA-B40 have also been shown in AS and it is noteworthy that ERAP-1 is involved in peptide trimming for T-cell presentation. RUNX3 SNPs has been reported in AS, combined with epigenetic regulation at the RUNX3 locus in CD8+ T-cells, which affects their differentiation in AS (51, 52). Thus, MHC-I, ERAP-1, and RUNX3 SNPs in AS strongly incriminate antigen presentation and adaptive CD8+ T-cell responses in disease pathogenesis (53, 54). Recent structural biology results showed that AS-associated HLA–B^*^27 subtypes B^*^27:04 and B^*^27:05 possessed elevated molecular dynamics compared to the non-associated sub- types B^*^27:06 and B^*^27:09 which further incriminates classical peptide presentation to cytotoxic T-cells (55).

However, unlike the MHC-I associated disease psoriasis, where putative autoantigens have been defined, specific antigens, either self, or foreign that drive disease have not been defined (56). This remains the biggest black box to be elucidated in early AS disease pathogenesis.

The failure to define classical adaptive immune responses in early AS, despite the growing indirect evidence for such a mechanism has resulted in research into alternative mechanisms. CD4+ T-cells are also thought to play a role in AS with CD4+ T-cells capable of recognizing and binding to misfolded HLA-B27 (57). Misfolding of HLA-B27 proteins with an unfolded protein response and ER stress also leads to macrophage activation with increased IL-23 induction, supporting a potential link between HLA-B27 misfolding and immune dysregulation (58). However, whilst AS HLA-B27 blood derived macrophages have been shown to secrete increased IL-23 when compared to healthy patients, it is thought this is independent of ER stress (59). Moreover, it was reported that dimers of HLA-B27 have been shown to promote survival of subsets of natural killer (NK) and CD4+ Th17 T-cells expressing KIR3DL2 (60).

## Basic and translational cytokine immunology of AS

The role of TNF-α in experimental SpA models that have oligoarthritis and sacroiliitis is well-established and these models are mechanically dependent (61). The normal human spinal entheses harbor type 3 ILCs IL-23R expressing cells, capable of IL-17 production, and are therefore also potential mediators of IL-23 driven enthesitis (62). However, although monocytes and macrophages are known to produce IL-23, the source of IL-23 acting at the enthesis remains to be fully elucidated but evidence for local myeloid production has been reported (63). Regarding immune cells and the previously mentioned IL-23/IL-17 axis, IL-17 producing ILC3 cells are more prevalent in the gut of AS patients when compared to healthy controls, with the highest correlation pointing toward the inflamed gut of AS patients (64). This adds weights to the growing theory of a possible gut-bone axis and a gut-inflammation-spondylitis link, with both IL-23/IL-17 and HLA-B27 acting as central players (65). The interaction between HLA-B27 and the IL-23/IL-17 is incompletely understood but may be linked to activation of Tc17 cells (CD8+ T-cells that produce IL-17) (66, 67). However, it has also been suggested that classical peptide presentation by HLA-B27 activates myeloid dendritic cells that subsequently drive a Tc17 adaptive immune response that is antigen dependent (12). We favor this classical model which was also touted and reinvigorated following the discovery of ERAP-1 SNPs in AS, especially given that this ERAP-1 is key to peptide presentation via HLA-B27 and other class-I antigens to T-cells.

## Emergent translational insights into early AS

IL-17 production that is independent of IL-23 is potentially crucial in the maintenance of AS (68) and IL-23 inhibition has no clear clinical benefit in established AS (69). Stromal cells have been shown to mediate the inflammatory response to mechanical stress (61) and have been shown to directly stimulate Th-17 production of IL-17 in an IL-23 independent manner by the release of prostaglandin E2 (PGE2) (70). In the gut, PGE2 is expressed constitutively and is crucial for homeostasis.

The γδ T-cells group form a multifaceted subset of cells that have a crucial role in gut homeostasis and have also been shown to produce IL-17 independently of IL-23 (71). There is a growing body of evidence to suggest that different entheseal cell populations such as γδ T-cells are key players in SpA pathogenesis and this may be IL-23 dependent or independent but the potential contribution of multiple cell types cannot be ignored. Recently it was experimentally shown that IL-23 is essential for the initiation of SpA, but not disease progression since the triggering of IL-17A and IL-22 resulted in independent disease progression (68). It has also been reported that macrophages from AS patients have a greater IL-23 secretory ability (59, 72). IL-17 producing cells have been confirmed in AS facet joints (73). Whilst the IL-23/IL-17 axis was thought to be once involved in both RA and SpA, clinical reports now suggest this axis is of more central importance in PsA and axial SpA such as AS (67). In the context of AS immunopathology, IL-22 may activate osteoproliferation (22). It should be emphasized that the majority of IL-17 studies in human AS have focused on established disease and not early stages, thus the relative importance of different immune pathways into disease initiation and progression are not known. In line with this point, it was is thus surprising that risankizumab a IL-23p19 inhibitor failed to show efficacy in AS (69), whereas secukinumab (anti IL-17) showed efficacy (74). Furthermore, the IL-12p40 blocking antibody ustekinumab, which also blocks IL-23 failed to show efficacy in 3 phase 3 clinical trials in AS, although the full results have not been published. However, a previous open-label study of ustekinumab in AS showed responses at week 24 (75). It is possible that IL-17 production in the spine that is independent of IL-23 signaling might be sufficient to drive disease, the opposite scenario to the gut where IL-17 production independent of IL-23 protects from disease, namely colitis. ILC3s are also emerging potential immune regulators of AS, capable of producing IL-17 and found to be expanded in the intestinal, synovial fluid, peripheral blood, and bone marrow of AS patients (64) and at the normal human enthesis (62).

## Lessons learned from animal model studies

Animal models have already been well-reviewed elsewhere and the focus of this article is on early human disease (76, 77). Transgenic mice and rats for HLA-B27 are known to develop spontaneous inflammatory diseases similar to humans such as SpA (78, 79). Other studies also highlight that HLA-B27 transgenic rats show an altered gut microbiota (80). A recent paper, using HLA-B27 rat model showed that spondyloarthritis pathology, is dependent on IL-23 for initiation but not persistence (68). This may indeed point toward the idea of IL-17 production that is independent of IL-23 in disease progression, whilst IL-23 is required for disease initiation. However, inhibition of IL-23 and IL-12 in dendritic cells in mice is shown to protect against T-cell immune mediated diseases (81). Different animal model studies of SpA are pointing toward a prominent role of local innate immunity originating due to local tissue injury as a component of tissue repair that in later phases leads to dysregulation of adaptive immunity (5). However, some models demonstrated an enhanced IL-17 production from naïve CD4+ T-cells, which are generated from a depleted number of migratory dendritic cells (82, 83). Th17 cells also promote the formation of IL-12/IL-23 producing dendritic cells, suggesting a cycling inflammatory pathway. IL-23 overexpression in mice causes a florid enthesitis which is driven by enthesis resident population of IL-23R positive innate T-cells that drove IL-17A dependent pathology (77). The primary source of IL-17 in this model were γδ T-cells rather than conventional αβ T-cells including Th-17 cells (84). The predominant subtype involved was the Vγ6 subset which has also been shown to invade sites of bone injury and contribute to repair via the production of IL-17 in an independent unrelated bone fracture model (85).

## Conclusion

We focused on the early phases of AS, where skeletal mechanical responses to stress and gut dysfunction, whether related to the microbiome, or specific microbes or simply intrinsic barrier or mucosal immune dysfunction are important. The TNF-α and the IL-17 pathways remain cardinal but unexpectedly, IL-23 does not have a role in established AS but may play a pivotal role in the early phases of AS. Further studies on early phases of AS are needed to elucidate different immunopathological, translational, and therapeutic aspects. The current immunology supports the concept of different innate immune cell populations being present in virtually all of the skeletal and extra-skeletal target sites of AS. Some of these cytokines clearly play a protective role in tissue homeostasis best exemplified for IL-17A in the gut. Other molecules including PGE2 are physiologically involved in tissue repair. The over exuberant repair responses and chronic bone formation phenotypes in established AS suggest that post inflammatory over exuberant repair responses might link dysregulated cytokine pathways leading to a new bone formation.

## Author contributions

AW, CB, TR, and RC participated in the design and writing of the manuscript. AW and DM participated in the design, coordination, and manuscript writing. AW developed the figure graphic design. HM-O has helped in drafting the revised version of paper and provided a critical review and approved the last version of manuscript.

### Conflict of interest statement

The authors declare that the research was conducted in the absence of any commercial or financial relationships that could be construed as a potential conflict of interest.
